# Search Term Identification Methods for Computational Health Communication: Word Embedding and Network Approach for Health Content on YouTube

**DOI:** 10.2196/37862

**Published:** 2022-08-30

**Authors:** Chau Tong, Drew Margolin, Rumi Chunara, Jeff Niederdeppe, Teairah Taylor, Natalie Dunbar, Andy J King

**Affiliations:** 1 Department of Communication Cornell University Ithaca, NY United States; 2 Department of Biostatistics School of Global Public Health New York University New York, NY United States; 3 Department of Computer Science & Engineering Tandon School of Engineering New York University New York, NY United States; 4 Jeb E Brooks School of Public Policy Cornell University Ithaca, NY United States; 5 Greenlee School of Journalism and Communication Iowa State University Ames, IA United States; 6 Cancer Control and Population Sciences Huntsman Cancer Institute Salt Lake City, UT United States; 7 Department of Communication University of Utah Salt Lake City, UT United States

**Keywords:** health information retrieval, search term identification, social media, health communication, public health, computational textual analysis, natural language processing, NLP, word2vec, word embeddings, network analysis

## Abstract

**Background:**

Common methods for extracting content in health communication research typically involve using a set of well-established queries, often names of medical procedures or diseases, that are often technical or rarely used in the public discussion of health topics. Although these methods produce high recall (ie, retrieve highly relevant content), they tend to overlook health messages that feature colloquial language and layperson vocabularies on social media. Given how such messages could contain misinformation or obscure content that circumvents official medical concepts, correctly identifying (and analyzing) them is crucial to the study of user-generated health content on social media platforms.

**Objective:**

Health communication scholars would benefit from a retrieval process that goes beyond the use of standard terminologies as search queries. Motivated by this, this study aims to put forward a search term identification method to improve the retrieval of user-generated health content on social media. We focused on cancer screening tests as a subject and YouTube as a platform case study.

**Methods:**

We retrieved YouTube videos using cancer screening procedures (colonoscopy, fecal occult blood test, mammogram, and pap test) as seed queries. We then trained word embedding models using text features from these videos to identify the nearest neighbor terms that are semantically similar to cancer screening tests in colloquial language. Retrieving more YouTube videos from the top neighbor terms, we coded a sample of 150 random videos from each term for relevance. We then used text mining to examine the new content retrieved from these videos and network analysis to inspect the relations between the newly retrieved videos and videos from the seed queries.

**Results:**

The top terms with semantic similarities to cancer screening tests were identified via word embedding models. Text mining analysis showed that the 5 nearest neighbor terms retrieved content that was novel and contextually diverse, beyond the content retrieved from cancer screening concepts alone. Results from network analysis showed that the newly retrieved videos had at least one total degree of connection (sum of indegree and outdegree) with seed videos according to YouTube relatedness measures.

**Conclusions:**

We demonstrated a retrieval technique to improve recall and minimize precision loss, which can be extended to various health topics on YouTube, a popular video-sharing social media platform. We discussed how health communication scholars can apply the technique to inspect the performance of the retrieval strategy before investing human coding resources and outlined suggestions on how such a technique can be extended to other health contexts.

## Introduction

### Background

Researchers are increasingly interested in understanding the types and accuracy of health-related messages produced in the public communication environment (PCE) [1-5]. Given the proliferation of web-based health information sources and social media platforms in which people generate, share, and access information [6], identifying and capturing what message content individuals are likely to see when looking for information about health (ie, seeking), as well as what information people might encounter while being on the web (ie, scanning) [7-9], is crucial in gaining insights into issues, including misinformation or inequities, on web-based platforms within the larger PCE.

Nevertheless, identifying appropriate strategies to retrieve this information is challenging. To gather data for analysis, researchers often rely on the standard approach of searching for content using keywords, which usually involve a set of technical (eg, medical) terms that describe a condition or behavior of interest (eg, “colon cancer” or “diabetes”) [10-12]. However, keyword search strategies that are solely based on technical concepts cannot account for the multifaceted nature of web-based information. A primary reason is that the messages in the contemporary PCE are often generated by users and, thus, often include colloquial terminology rather than medical terminology [7,13-15]. This phenomenon has been well documented in consumer health vocabularies research, which examines the language gap between official medical texts and user-generated content, such as question and answer (Q&A) sites (Yahoo! Answers) and social media platforms (eg, Twitter) [16-19].

In addition to messages that do not include technical keywords, another type of content that might be overlooked by the standard retrieval approach is what could be categorized as content that misleads by omission (eg, messages that describe risky behaviors but fail to name the medical risk it exposes an individual to) [20-22]. For example, messages promoting a fad diet, which might be associated with a specific medical condition but do not mention this risk nor the condition itself, will not be retrieved by keywords naming the condition.

Failure to retrieve these messages could result in the biased identification of content, especially in light of research showing how search results vary according to specific queries [23] and how social media language varies across different geographical locations [24]. In other words, retrieving (and analyzing) only messages produced with the “official” technical language can lead researchers to overlook the information consumed and barriers faced by underprivileged groups [25,26] or users who lack the skills and knowledge to correctly use official medical vocabularies to access information [27,28]. For these reasons, public health researchers trying to understand the PCE would benefit from a principled, replicable process for searching for web-based content relevant to medical terms but not exclusively restricted to them. Such a process would also inform web-based users’ health information–seeking efforts by enabling the retrieval of health-related information from commonly used slang or nontechnical queries.

This paper proposes such a retrieval process for YouTube. Using the platform’s application programming interface (API) to retrieve videos and the inferred relatedness between videos determined by YouTube’s proprietary algorithm, our process retrieves videos that (1) are frequently relevant to understanding the PCE related to a focal technical term, (2) are distinct from the videos retrieved directly with the focal term, and (3) can be easily distinguished from irrelevant videos that could otherwise absorb researchers’ attention. Such a search identification approach balances the trade-off between recall and precision [29], identifying content that would not have been found using typical keywords without requiring human coders to sift through large quantities of irrelevant content.

In the following sections, we summarize relevant research on PCE content retrieval, highlighting strengths and weaknesses. We then discuss the rationale for using YouTube before detailing the techniques used to identify relevant content beyond formal medical concepts. We illustrate the techniques using cancer screening as a case study. We conclude with a discussion of the potential for application of the technique across other topics and platforms.

### Challenges of Health-Related Vocabulary Inconsistencies

User-generated health content presents important challenges to researchers attempting to retrieve content from this environment, particularly as (1) researchers may not know the vocabulary users use to discuss health topics and (2) users can mislead each other by failing to mention relevant information.

Research has shown that patients often do not conceptualize diseases, treatments, or risks in the same terms as health care practitioners [30-32]. Most plainly, the literature on consumer health vocabulary [15-17] shows that the terms used by laypeople are different from those used by health care practitioners. For example, questions about health topics posted on Q&A sites (eg, Yahoo! Answers and WebMD) by laypeople were found to contain misspelled words, descriptions, and background information and were more colloquial than texts by health professionals [13,33]. A more recent example is the COVID-19 pandemic, where infodemiologists identified a variety of terms using Google Trends that referred to the virus, including “stigmatizing and generic terms” (eg, “Chinese coronavirus” and “Wuhan virus”) that had not been identified by other research using more agreed upon and technical language about the virus [34]. These works suggest that user vocabulary, which is distinct from medical vocabulary, is important for understanding how individuals conceive of their health and the medical vocabulary related to it when looking for or coming upon health information on the web. More broadly, these different terminologies can reflect different ways of conceptualizing health issues [32,35,36].

It is not surprising then that user vocabulary is important for identifying relevant health-related posts on social media, as research indicates that retrieval performance significantly changes when users’ health queries are reformulated using formal, professional terminologies [23]. Thus, if researchers do not know what the user vocabulary is for a given topic, their retrieval strategy will be biased to identify only content posted by users who use technical medical vocabulary. Moreover, this bias is unlikely to be neutral with respect to larger public health concerns. In particular, differences of this nature, such as conceptualization of illness and preferred vocabulary, have been shown to be associated with important differences in outcomes [25,26,37]. Such conceptual differences would likely manifest in differences in user vocabulary.

### Problems of Omission in Health Information Retrieval

Another weakness of retrieving user-generated health messages with technical terms is that this strategy cannot, by definition, identify information that omits that term. However, this failure to connect risks to outcomes can be precisely what makes user-generated content misleading. It is well established that many people lack broad knowledge about risk factors for many leading causes of death in the United States and beyond [38-40], and people routinely receive information that fails to link common risk factors and behaviors to negative health outcomes [41]. Perhaps the best known (and most damaging) example is the failure of tobacco companies to mention that cigarette smoking causes cancer in their promotional materials [42]. This misrepresentation by omitting and distancing from medical terms (eg, disease) is common for unhealthy products (eg, alcohol) [43].

In such cases, the PCE misleads by omission as it fails to assign the appropriate words to what is medically accurate in the offline world. This has the potential to mislead the public and makes relevant messages hard to find, as their relevance (to researchers) is defined by what is absent (the mention of the risk). An example is the “Tide Pod challenge” that emerged in 2017 as a popular internet trend. The Tide Pod challenge is dangerous as it fails to connect the terms “Tide detergent” and “eat” with the concept (or concept family) of “poison.” A trained medical professional would not discuss “eating” Tide Pods without also mentioning the danger, although users can (and did) do so. Such misleading (and dangerous) user messages cannot be retrieved by strategies that focus on the harm—poisoning.

In the case of well-researched and widely understood risks, such as the connection between cigarette smoking and lung cancer, this weakness can be overcome by simply naming the risk factor (ie, searching for “lung cancer”). However, to restrict searches to known and well-documented high-risk behaviors is to again return researchers to their cultural bubble [44]. As evidenced by the emergence of the Tide Pod challenge, user-generated content can be extraordinarily inventive, creating new risky behaviors unknown to the medical community. For example, dangerous fad diets cannot be identified by searching for the risks they pose. Instead, what is needed is a way of identifying vocabulary that is “near” to the condition of interest, broadening the net so that researchers can identify messages misleading by omission.

For both reasons, researchers should find ways to escape the strictures of official, technical vocabulary when retrieving information to characterize the PCE. Researchers instead need search terms that include culturally relevant colloquial terms that are related to medical terms and terms that identify behaviors or practices *in the neighborhood* of medical terms but which can identify content when those terms are omitted.

### YouTube as Public Health Information Source and Site of Inquiry

In this study, we focus on YouTube videos as a meaningful message source of the PCE. We selected YouTube for 2 reasons. First, YouTube is one of the most widely used web-based social media and content platforms [45]. Second, YouTube has become increasingly relevant as a source of health information. With its dual function as a reservoir of video content and a social networking platform in which users acquire information through interactions with the content and fellow users, YouTube has served as an informational resource for learning about diverse health topics for users [46,47].

Extant research on medical and health information on YouTube suggests several issues with the quality of YouTube content. A meta-analysis found that YouTube videos tend to prevalently contain misinformation, an implication of which is the potential of the platform to alter beliefs about health interventions [46]. A limitation of these studies (and a weakness shared by many YouTube studies) is the search strategies used to identify relevant content. To address this gap in current research, our project aims to answer 2 research questions (RQs).

The first main RQ asks the following: for a given medical or health term of interest (ie, a focal term for retrieval), does our proposed search term identification strategy retrieve health messages that are relevant to understanding the public health communication environment related to that seed term and do not explicitly use that term (such that the traditional medical or technical search terms would have failed to retrieve them)? To provide a satisfactory answer to this question, a search strategy must (1) retrieve content relevant to the seed term (called precision) and (2) find relevant content that is novel, (ie, different from what would be returned by the seed term alone, called recall), without sacrificing too much precision. This leads to our second RQ: can the derived strategy identify relevant, novel messages with sufficient precision to be practically useful?

## Methods

### Rationale for Cancer Screening Focal Terms

Cancer is one of the biggest public health issues in the United States and, thus, is a topic that requires meticulous attention from multiple stakeholders, including public health practitioners and communicators. A particular challenge to the prevention and management of various cancer types is the persistent disparities in screening, incidence, and mortality rates across different population groups [48]. Given the significance of cancer and the important implications of cancer screening disparities, we chose cancer screening as the subject of examination in this paper.

To this end, we first demonstrate our methodological technique using the primary colorectal cancer screening option—“colonoscopy”—as our focal term. Colorectal cancer is the third most diagnosed and third most deadly cancer in the United States, which disproportionately affects Black individuals compared with non-Hispanic White Americans [49]. We then replicate the analyses using other cancer screening tests (fecal occult blood test, mammogram, and pap test) as focal terms to illustrate how the technique performs in other cancer contexts, including breast and cervical cancer.

### Retrieving YouTube Videos From the Focal Term

We collected data from YouTube via the YouTube API (version 3). Using the “search: list” end point (used for the search function) allowed us to retrieve 2 types of data: videos that are most relevant to a search query or set of queries (the “q” parameter with “relevance” sorting) and videos that are related to a specific or set of videos (the “related-to-video-id” parameter) according to YouTube algorithms [50]. We note that collecting data through this API approach bypasses localization and personalization—factors that play important roles in search results that are presented to specific individuals. As our purpose is to demonstrate a methodology that can be systematically extended to other contexts in future research, we deem this approach to be appropriate in giving us the results as close to a default setting as possible.

On August 22, 2021, using the YouTube Data Tools software [51], we retrieved a set of 250 videos most relevant to the search term “colonoscopy.” These 250 videos comprise our core set. In addition, we retrieved 4304 videos “related to” this core set, which gave us 4554 videos in total in the initialization set. We retrieved these videos’ unique identifiers, text data (video titles and descriptions), and metadata (publication date and engagement statistics).

### Word Embeddings

Word embedding is an unsupervised method of learning word vectors using a neural network model [52]. The basic aim of word embeddings is to identify words that appear in “similar contexts” as the focal term. The technique calculates a proximity score; that is, the extent to which 2 terms are near to one another in a multidimensional space. This score acts as a measure of “semantic similarity.” Thus, it is a useful way of finding texts that discuss a particular concept without explicitly mentioning it. Texts that mention a word’s close neighbors (in the multidimensional space) are likely talking about ideas where that word is relevant as well, even if the word itself is not there. We used word embeddings to find YouTube content that is relevant to “colonoscopy” but which may not mention the word itself.

We applied word embeddings using the word2vec approach to the text data of our initialization set of 4554 videos. Specifically, we used the text of the 4554 video titles and descriptions to build a corpus. Subsequently, after preprocessing and standardization steps (including removal of emojis, signs, and stop words; performing lowercasing; converting text to American Standard Code for Information Interchange; encoding; and removing leading or trailing spaces), a word2vec model was trained on the text to identify the terms with the most semantic similarity to the term “colonoscopy” (word2vec R package) [53].

We then used the top 6 “nearest neighbors” to “colonoscopy” as new search terms to retrieve more videos (250 videos for each neighbor) to inspect the new content.

### Human Coding and Natural Language Processing to Evaluate Recall Improvement

The goal of retrieving new content from the nearest neighbors is the improvement of recall over a direct search—the identification of videos that are relevant to “colonoscopy” but which would not be found by searching directly for it. To assess this recall improvement, we took a random 10% (150/1500) sample (25 videos for each neighbor) and coded them for relevance. Coding was done by a research team member (AJK, the paper’s last author) with expertise in cancer control and cancer communication.

Specifically, a video was coded as relevant if the video content contained (1) any aspect of screening preparation or procedures (eg, bowel preparation, personal experiences, and clinical discussions) or (2) general information on colorectal cancer or colorectal cancer screening in terms of cancer prevention or early detection. This included content where a patient underwent a colonoscopy but perhaps for a chronic condition (eg, ulcerative colitis or Crohn disease). Obscure terms identified through this process were also looked up as needed to confirm relevance (eg, “suprep”—a commercial brand for a bowel preparation kit).

We evaluated recall in 2 ways. First, we assessed how many of the relevant “found” videos would have been identified using the search term alone. We did this by counting the number of relevant videos in the newly found set containing the term “colonoscopy.” Those that did not contain “colonoscopy” but were nonetheless relevant to it constituted a recall improvement. Second, we examined whether these newly found videos were substantively different—in terms of contents, topics, and focus—from the core set. Using the R package *quanteda* [54], we calculated the average Euclidean distances between the text features embedded in the different video sets. Euclidean distance is a pairwise distance metric that measures dissimilarities between the text features in different corpora. We then used hierarchical clustering analysis, with the complete linkage method (*hclust* function in *stats* version 3.6.2), to determine whether videos in different sets were substantially overlapping in content.

### Network Analysis to Evaluate Precision

Strategies to improve recall are often offset by a substantial loss of precision. In our case, although the nearest neighbors may retrieve many more relevant videos, they could, at the same time, bring in many irrelevant videos. This introduces the risk of increasing human coding costs or other resource-intensive techniques of classification. Such precision loss needs to be mitigated so that it occurs at a manageable level. To implement this, we used the “related to video id” API end point, which reports whether a set of videos are “related to” the others (zero crawl depth), to query the relationships between the new videos retrieved from the top neighbor terms and the colonoscopy videos from the core set. Specifically, if video A is related to video B in a set, there is a connection (or link) between them. These relations were used to create a network with videos being nodes and the connections between them being edges.

We then calculated 3 network measures of relatedness: indegree (videos from the core set linking to a newly found video), outdegree (videos in the core set linking to each newly found video), and total degree (sum of indegree and outdegree). We expected that the newly found irrelevant videos would have few, if any, links to the videos known to be about “colonoscopy,” whereas videos with even loose relevance would have at least some connections to the core set. To examine the extent to which these degree scores were associated with relevance (according to human coding), the corresponding precision and recall statistics at different degree levels were inspected. If our technique worked effectively, there would be some threshold of degree—the number of connections between a newly found video and the core set—at which videos with this degree or higher are not only reasonably novel (improving recall over the core set) but also reasonably relevant (maintaining precision at a manageable level).

### Ethics Approval

This study did not involve the use of human subjects, as the data collected were strictly limited to publicly available data on YouTube; therefore, no ethics approval was applied for. This rationale is consistent with the institutional policies where the research was conducted.

## Results

### Word Embeddings

Table 1 provides the list of neighbor terms to the focal term “colonoscopy” and their ranks based on semantic similarity, according to word embedding results.

A visual inspection suggests these nearest neighbor terms fit our goals for this method: they contain nontechnical terms (eg, “cleanse” or brand names such as “plenvu”) that are relevant to colorectal health. We selected the top 6 terms (“suprep” to “miralax”), retrieved an additional 1500 videos (250 each), and coded a subset of 10% (150/1500 random videos) for the recall analysis.

**Table 1:** Neighbor terms to “colonoscopy” and similarity scores.

Term^a^	Similarity score	Rank
“suprep”	0.9722890	1
“peg”	0.9519246	2
“sutab”	0.9513488	3
“plenvu”	0.9504289	4
“glycol”	0.9498276	5
“miralax”	0.9449067	6
“rectal”	0.9435940	7
“cleanse”	0.9422708	8
“cologuard”	0.9421358	9
“colorectal”	0.9403084	10

^a^Neighbor terms are terms with the most semantic similarity (with corresponding high similarity scores or low ranks) to “colonoscopy” based on YouTube video data. Score refers to the cosine similarity metric between word embeddings (ie, terms) in a multidimensional vector space.

### Human Coding and Natural Language Processing to Evaluate Recall Improvement

Table 2 displays the retrieval statistics, of which 34% (51/150) of the coded videos were deemed relevant. More importantly, of these 51 videos, 21 (41%; 21/150, 14% of the coded sample) did not contain the term “colonoscopy,” meaning that identifying them improved recall over what would have been found simply by searching for “colonoscopy.” This supported our expectation that the word embedding approach helped address the recall problem inherent in using technical language.

We next assessed whether these newly found videos were substantively different—in terms of contents, topics, and focus—from what would be retrieved with the typical strategy. To assess this, we compared the Euclidean distances between textual features of the core set (250 videos) with those of the newly found videos (Table 3). Here, higher values meant greater distance. For example, the distance between “miralax” and “peg” was the smallest among our groupings, indicating that videos in these 2 sets shared the most similar words compared with other pairs.

**Table 2:** Retrieval statistics in the sampled videos for the top 6 neighbors of “colonoscopy.”

Terms	Sample of coded videos, N	Relevant (precision), n (%)	Relevant and mention of “colonoscopy,” n (%)	Relevant and does not mention “colonoscopy” (recall improvement), n (%)
“suprep”	25	18 (72)	9 (36)	9 (36)
“peg”	25	1 (4)	0 (0)	1 (4)
“sutab”	25	4 (16)	4 (16)	0 (0)
“plenvu”	25	23 (92)	15 (60)	8 (32)
“glycol”	25	0 (0)	0 (0)	0 (0)
“miralax”	25	5 (20)	2 (8)	3 (12)
Total	150	51 (34)	30 (20)	21 (14)

**Table 3:** Euclidean distance between the text features of original “colonoscopy” video set and video sets generated from top 6 neighbor terms^a^.

Term	1	2	3	4	5	6
“colonoscopy”	0	255.61	257.97	241.5	248.9	254.68
“miralax”	N/A^b^	0	6.32	20.1	21.8	7.14
“peg”	N/A	N/A	0	22.2	23.1	6.86
“plenvu”	N/A	N/A	N/A	0	20.6	19.08
“suprep”	N/A	N/A	N/A	N/A	0	20.57
“sutab”	N/A	N/A	N/A	N/A	N/A	0

^a^Cell values indicate dissimilarities of the text features belonging to any pair of video sets. Larger values indicate larger distances, and 0 indicates identical text features. “Glycol” was removed because of 0 relevant videos retrieved.

^b^N/A: not applicable.

**Figure 1:**
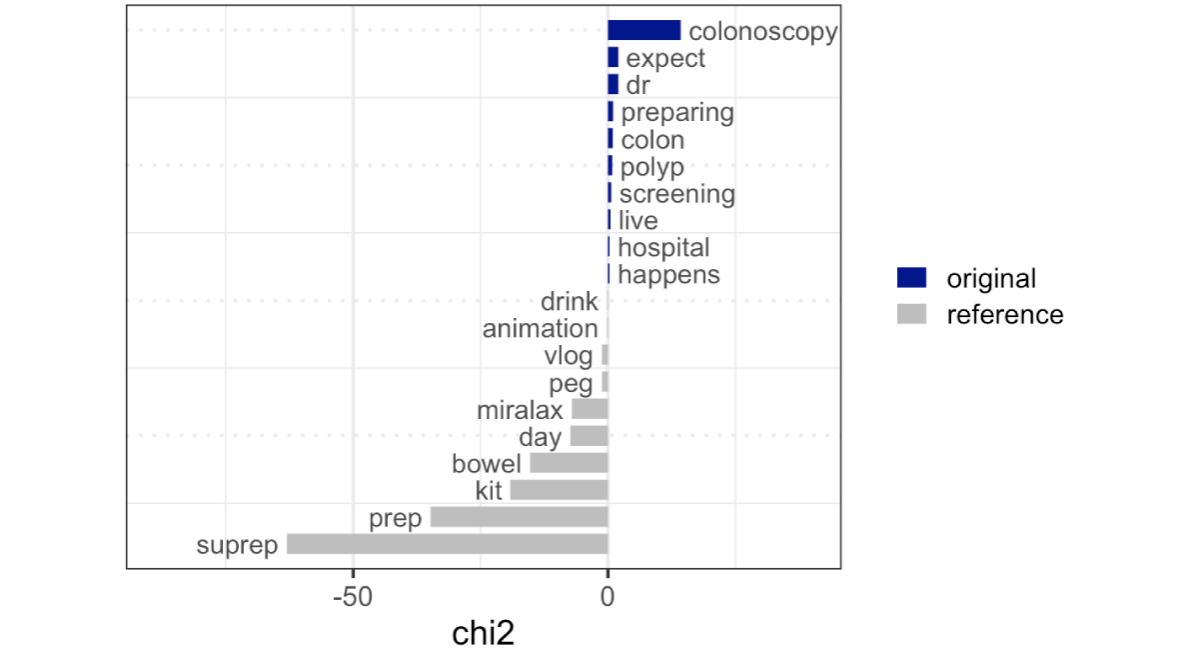
Relative frequencies of words in the colonoscopy video set and the combined top 5 neighbor term video set. Words that are “key” to each video set were plotted. Original: the set of videos found with the search query “colonoscopy.” Reference: the set of videos found with 5 nearest terms to “colonoscopy” (“suprep,” “peg,” “sutab,” “plenvu,” and “miralax”). chi2: chi-square value.

Relative frequency analysis was used to further illustrate these differences by highlighting the differences in the text features of the core set as opposed to the newly found set. As Figure 1 shows, words such as “colonoscopy,” “dr,” “preparing,” “colon,” and “polyp” were disproportionately more likely to occur in the core set, whereas words such as “suprep,” “prep,” “kit,” “bowel,” and “miralax” were distinct terms found in the newly found set.

**Figure 2:**
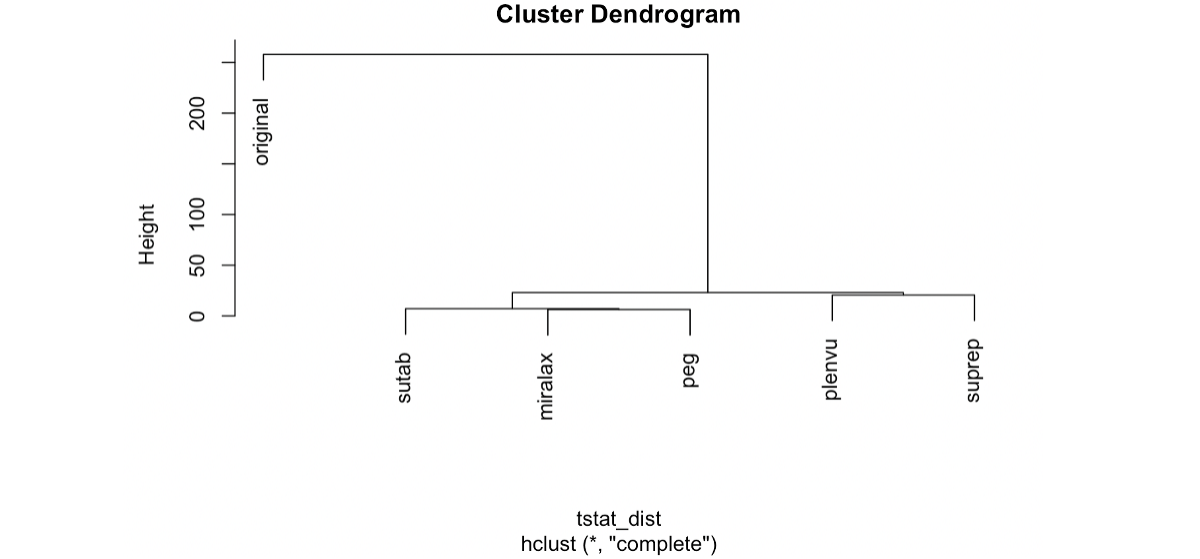
Visualization of distances between video sets. Hierarchical cluster analysis indicating dissimilarities and distances between original (set of videos found with the search query “colonoscopy”) and sets of videos found with 5 nearest terms to “colonoscopy” (“suprep,” “peg,” “sutab,” “plenvu,” and “miralax”).

Hierarchical agglomerative clustering performed on the text features of the newly found set and the core set (using the complete link method) revealed that the text features in the videos retrieved from neighbor terms (newly found set) were more similar to such from other neighbor terms than to the core set (Figure 2). In other words, these results show that our approach helped identify videos that are relevant to “colonoscopy” without including the term itself (ie, improving recall); furthermore, these newly found relevant videos additionally enhanced the topical diversity of our retrieved data (by focusing on preparation brands and procedures).

### Network Analysis to Evaluate Precision

Table 4 shows the results of the comparison between a found video’s degree of connection to the core set and its associated relevance according to human coding. We first note that new videos that are in other languages than English (28/150, 18.7%) were found to have no connections with the core set videos. To avoid having this add bias to our results, we excluded these 28 videos, as well as 8 videos that were already found in the original set and 1 video where YouTube returned missing metadata (37/150, 24.7% excluded in total). We then performed a comparison on the remaining 75.3% (113/150) of videos (the final total in the “cumulative count of videos,” also the denominator).

**Table 4:** Relevance of newly found videos by the number of links to the original set of colonoscopy videos (total degree).

Total degree^a^	Count of videos with total degree, N	Number of videos coded as “relevant” (relevancy), n (%)	Cumulative count of nonduplicate videos, N	Cumulative count of nonduplicate relevant videos, n	Cumulative precision^b^ (%)	Cumulative recall^c^, (%)	Cumulative *F*_1_-score^d^, (%)
44	1	1 (100)	1	1	100	2.7	5.3
41	1	1 (100)	2	2	100	5.4	10.3
26	1	1 (100)	3	3	100	8.1	15.0
23	1	1 (100)	4	4	100	10.8	19.5
22	1	1 (100)	5	5	100	13.5	23.8
21	1	1 (100)	6	6	100	16.2	27.9
20	2	2 (100)	8	8	100	21.6	35.6
19	1	1 (100)	9	9	100	24.3	39.1
18	1	1 (100)	10	10	100	27.0	42.6
17	2	2 (100)	12	12	100	32.4	49.0
16	1	1 (100)	13	13	100	35.1	52.0
15	2	2 (100)	15	15	100	40.5	57.7
14	1	1 (100)	16	16	100	43.2	60.4
13	1	1 (100)	17	17	100	45.9	63.0
12	2	2 (100)	19	19	100	51.4	67.9
11	2	2 (100)	21	21	100	56.8	72.4
10	1	1 (100)	22	22	100	59.5	74.6
9	1	1 (100)	23	23	100	62.2	76.7
7	2	1 (50)	25	24	96	64.9	77.4
6	1	1 (100)	26	25	96	67.6	79.4
5	2	0 (0)	28	25	89	67.6	76.9
4	2	2 (100)	30	27	90	73.0	80.6
3	2	1 (50)	32	28	88	75.7	81.2
2	5	1 (20)	37	29	78	78.4	78.4
1	5	1 (20)	42	30	71	81.1	75.9
0	71	7 (10)	113	37	33	100	49.3

^a^The sum of connections each new video has with the videos in the original colonoscopy video set.

^b^The cumulative count of relevant videos divided by the cumulative count of all videos.

^c^Cumulative count of relevant videos divided by the total number of new and nonduplicate 37 relevant videos.

^d^The harmonic mean of cumulative precision and cumulative recall.

The first 4 columns in Table 4 show the total degree (number of connections) and counts of videos with corresponding total degrees in comparison with the relevance statistics. Specifically, all videos with a total degree >7 had been coded as relevant, meaning precision is 100% at or above this threshold. More importantly, although precision was imperfect below this threshold, it remained very high. In fact, when we examined videos of degree ≥1, we found that 71% (30/42) had been coded as relevant. This means that a human coding team choosing to use this liberal threshold (at least one connection to any video in the core set) for choosing videos to code would see >2 relevant videos for every irrelevant one, thus expending limited resources examining irrelevant videos.

The cumulative columns on the right-hand side of the table display the trade-offs that would face a coding team. The cumulative count of relevant videos adds up to 37, which is the 51 coded as relevant (Table 2) excluding the 8 videos already found in the original data set (as reported above) and 6 non-English videos that had been coded as relevant. Cumulative precision refers to the relevance of the videos at or above this threshold. Cumulative recall shows the portion of the relevant videos in the set that are preserved at this threshold. As the threshold tightens, precision improves (irrelevant videos are discarded) but recall declines (some relevant videos are discarded too). For example, if a team chose to examine videos with at least three connections to the core set (degree ≥3), they would find 32 videos, 28 of which are relevant (88% precision), and miss out on only 9 of the 37 possible (75.7% recall). In other words, this technique provides a basis for researchers to inspect the performance of the retrieval strategy before investing human evaluation and coding resources.

### Replication: Other Cancer Screening Tests

We extended our analyses to 3 additional focal terms to illustrate the breadth of the technique’s applicability. The first, “FOBT,” refers to the fecal occult blood test, another screening method for colorectal cancer. The second and third are “mammogram” and “pap test,” screening tests for breast cancer and cervical cancer, respectively. We chose cancer screening as an illustrative case as these are common cancer types that are often discussed on social media [3,55] such that research would benefit from identifying relevant content that does not explicitly mention these technical, formal screening tests.

As shown in the summary statistics in Table 5, the results for these terms were comparable with “colonoscopy.” For each focal term, searches using the nearest neighbor terms uncovered through word2vec identified a wide range of new videos that were distinct from the original sets, improving recall (see Multimedia Appendix 1 for dissimilarity measures of new vs original content). Similar to the results for “colonoscopy,” filtering videos based on their degrees of connection to the core set (for the respective focal term) improved precision while maintaining reasonable recall. For both “FOBT” and “pap test,” researchers could inspect only videos with a degree of ≥1 and would find a few irrelevant videos while maintaining most of the new videos in the set. For “mammogram,” the recall statistics of videos with at least one connection is lower (30%); however, even if researchers chose to drop this filter and inspect all videos, they would find that approximately 1 in 3 new videos found is relevant. Thus, researchers would not be at risk of being overwhelmed with irrelevant content.

**Table 5:** Summary retrieval statistics for “colonoscopy,” “FOBT,” “mammogram,” and “pap test.”

Focal term	Top nearest neighbor terms	Sample of coded videos (videos per term)	New and nonduplicate relevant videos (set A), N	Videos with degree ≥1 (set B)^a^, N	Videos with degree ≥1^a^ and coded as new and relevant, n (A∩B)	Precision, n/N (%)	Recall, n/N (%)
Colonoscopy	“suprep” “peg” “sutab” “plenvu” “glycol” “miralax”	150 (25)	37	42	30	30/42 (75)	30/37 (81)
FOBT^b^	“iFOBT” “hemosure” “immunochemical” “immunostics” “guaiac”	125 (25)	50	33	27	27/33 (82)	27/50 (54)
Mammogram	“smartcurve” “breastcheck” “biopsy” “ultrasound” “breastcancerawareness”	250 (50)	77	28	23	23/28 (82)	23/77 (30)
Pap test	“Colposcopy” “Smear” “ASCUS”^c^ “papsmear” “STD”^d^	250 (50)	87	65	59	59/65 (91)	59/87 (68)

^a^Videos with at least one connection to the original set of videos resulted from the focal terms.

^b^FOBT: fecal occult blood test.

^c^ASCUS: atypical squamous cells of undetermined significance.

^d^STD: sexually transmitted disease.

## Discussion

### Principal Findings

This paper proposes a novel approach to improving the retrieval of user-generated health content. Using medical concepts as focal terms, we used the similarity-based word embedding approach to detect new search terms related to focal terms but not restricted to technical vocabulary. In line with previous research using similar methods (eg, word, sentence, or biomedical term embeddings), we identified less widely known terms in user-generated public discourse related to cancer screening tests. Quantitative textual analysis of the newly discovered content returned from the top neighbor terms indicated that these videos were distinct from the original video sets in terms of lexical and topical foci. Network analysis showed that retrieval precision can be improved by detecting videos with at least one total degree; that is, those with at least one connection to others in the same networks. Researchers could use the technique to inspect the performance of their retrieval strategy before investing additional evaluation resources [56,57]. Beyond suggesting the value of this technique, our analyses provide insights into specific message gaps if user-generated vocabulary is overlooked.

First, our results indicate that commercial speech, particularly tagged by brand names such as “suprep” and “miralax,” was particularly prominent and useful for identifying relevant content. In essence, users produced and consumed videos about “prepping,” which could be used for colonoscopies, in reference to branded products. This raises an important follow-up question—do these videos provide accurate information? As reviewed previously, the history of corporate actors misleading consumers by omission of risks is substantial [58,59]. Although this would be an analysis for further study, we point out here the importance of retrieving information about medical topics using commercial terms rather than just medical or technical terms.

Second, we note that our results did not provide examples of de novo slang synonyms (akin to “the sugars”). Rather, when users created terms, they were more likely to be portmanteaus of simple vocabularies, such as “breastcheck,” “papsmear,” or even “breastcancerawareness.” This merging of words into one term is unsurprising insofar as it is consistent with the conventions for the creation of hashtags; however, this should serve as a caution to researchers to consider these nonstandard constructions in their retrieval strategies. In other words, for the terms searched in this study, we found little evidence of colloquial language. However, for any health topic, there is the possibility that such language is used in less intuitive ways. Although we did not find that to be the case for our focal terms, the possibility exists, and this technique could have the potential to identify such in other cases.

More broadly, our analysis reveals that although user-generated vocabulary can often be sensibly interpreted after the fact (Plenvu’s website advertises it as a colonoscopy prep technique, and “breastcheck” is intuitively related to breast cancer), the most common terms are not always easy to guess in advance, that is, before analyzing some data. This observation supports the arguments that motivated this research, suggesting that researchers should first learn how users talk about medical topics and then create retrieval strategies to build fuller data sets for analysis of what they are saying. Although we do not have explicit evidence here that vocabularies are associated with particular social groups, or, in particular, marginalized groups, the presence of corporate brand names suggests, at the very least, that targeted marketing efforts could play such a role for particular medical topics. This is a topic for further research.

### Limitations

There are several limitations to this study. First, our analysis focused only on cancer screening tests as focal terms because of this project’s inclusion in a larger project focusing on colorectal cancer screening information in the PCE. Our purpose was to demonstrate a methodological technique in the context of cancer with the understanding that future research will need to assess any unique challenges that might apply to noncancer screening health topics or medical terminologies of interest (eg, vaccines or information about diabetes management). Although we see no methodological reasons why this technique could not be applied to other keywords and terminologies, future research would be needed to support this expectation.

The second limitation is that the word embedding model was trained on YouTube textual content, and our technique relied on YouTube’s relatedness data to distinguish between relevant and irrelevant videos. This means that the effectiveness of the present approach is limited to YouTube. Although there are good reasons to start with YouTube as a prevalent source of health-related information, we encourage future research to consider developing similar approaches for other domains where user-generated texts are found on the web, including websites, Q&A forum posts, and other social networking sites [21,57]. Importantly, many specific techniques may not be exportable from platform to platform. For example, although YouTube tracks relatedness between videos, messages on Twitter are often related by hashtags. Thus, rather than searching for relevant neighbor words, researchers might focus on identifying relevant neighbor hashtags. In Q&A forums or other content with threaded replies, researchers might incorporate this hierarchical information to identify the most relevant content (eg, terms used in top-level posts).

A final limitation is that conducting this process requires some familiarity with available natural language processing and computational tools. We believe the increasing application of computational methods in social science research, as well as the proliferation of training in R and Python languages for social scientists, increases the likelihood that this technique could be used by those with limited natural language processing proficiency. Nevertheless, health communication is an inherently interdisciplinary field in which we see great potential for collaborations among communication scientists, public health and medical researchers, and data scientists. However, future work might strive to make this technique more accessible through the creation of specific tools and materials to assist health communicators and public health professionals in applying these approaches in future health promotion and education efforts.

### Conclusions

This study demonstrated the potential of using similarity-based word embedding techniques for computational health communication research to improve recall and maintain precision in retrieving content that could be overlooked by standard medical terminologies. The study reveals that there are indeed relevant messages to medical topics in the PCE that do not use medical vocabulary, and that many of these can be identified. Although the impact of overlooking these messages on health disparities cannot be determined, these results suggest that further study in this area is warranted.

## Appendix

Multimedia Appendix 1 Supplementary replication analysis.

## Abbreviations

API: application programming interface
PCE: public communication environment
Q&A: question and answer
RQ: research question
