# Supplementary material for: Search Term Identification Methods for Computational Health Communication: Word Embedding and Network Approach for Health Content on YouTube
Source: JMIR Med Inform. 2022 Aug 30;10(8):e37862. doi: 10.2196/37862 (PMC9472050; doi:10.2196/37862)
Supplement: Multimedia Appendix 1 [file medinform_v10i8e37862_app1.docx]

# **Supplementary Replication Analysis**

Each table below reports the detailed Euclidean distance results between the text features of original video set for “FOBT”, “mammogram,” and “pap test,” and video sets generated from their own’s top neighbor terms

|  | Term | 1 | 2 | 3 | 4 | 5 | 6 |
| --- | --- | --- | --- | --- | --- | --- | --- |
| FOBT | - “FOBT” | 0 | 522.06 | 522.74 | 517.61 | 522.21 | 522.82 |
|  | - “iFOBT” | — | 0 | 7.14 | 10.30 | 9.11 | 9.27 |
|  | - “hemosure” | — | — | 0 | 9.95 | 7.87 | 7.28 |
|  | - “immunochemical” | — | — | — | 0 | 8.89 | 11.05 |
|  | - “immunostics” | — | — | — | — | 0 | 7.94 |
|  | - “guaiac” | — | — | — | — | — | 0 |

|  |  | 1 | 2 | 3 | 4 | 5 | 6 |
| --- | --- | --- | --- | --- | --- | --- | --- |
| Mammogram | - “mammogram” | 0 | 247.9 | 241.8 | 247.8 | 249.1 | 243.3 |
|  | - “smartcurve” | — | 0 | 23.7 | 16.3 | 11.4 | 43.7 |
|  | - “breastcheck” | — | — | 0 | 22.7 | 24.3 | 36.2 |
|  | - “biopsy” | — | — | — | 0 | 13.5 | 42.6 |
|  | - “ultrasound” | — | — | — | — | 0 | 45.2 |
|  | - “breastcancerawareness” | — | — | — | — | — | 0 |

|  |  | 1 | 2 | 3 | 4 | 5 |
| --- | --- | --- | --- | --- | --- | --- |
| Pap test^[[1]](#footnote-1)^ | - “pap test” | 0 | 690.1 | 688.27 | 691.88 | 668.2 |
|  | - “colposcopy” | — | 0 | 38.94 | 38.91 | 49.3 |
|  | - “smear” | — | — | 0 | 9.38 | 30.3 |
|  | - “ASCUS” | — | — | — | 0 | 35.4 |
|  | - “papsmear” | — | — | — | — | 0 |

1. “STD” was removed due to zero relevant videos retrieved. [↑](#footnote-ref-1)
